# Supplementary material for: Comparison of laparoscopic tubal-preserving surgical management versus methotrexate therapy for tubal pregnancy: A conventional and network meta-analysis
Source: PLoS One. 2025 Sep 12;20(9):e0332169. doi: 10.1371/journal.pone.0332169 (PMC12431245; doi:10.1371/journal.pone.0332169)
Supplement: Attachment — Submitted filename: Journal review.docx [file pone.0332169.s002.docx]

**Journal review**

Comparison of tubal-preserving surgical management versus methotrexate therapy for tubal pregnancy: a conventional and network meta-analysis

Tubal pregnancy is one of the common emergencies in obstetrics and gynecology. There are various treatment options for tubal pregnancy. However, there is no consensus on how patients should choose among the treatment options that preserve the fallopian tube.

**Result**

A total of 8 studies involving 677 patients were included. Meta-analysis revealed a significant overall difference in the failure rate between methotrexate treatment and salpingostomy for tubal pregnancy(OR=1.582, 95%CI(1.062, 2.358), p = 0.024). Subgroup analysis demonstrated that a single dose of methotrexate was associated with a higher failure rate compared to salpingotomy (OR = 2.044,95%CI(1.20, 3.47), p = 0.008). In contrast, two or more doses of methotrexate did not show a significant difference in failure rate compared to salpingotomy (OR = 1.130, 95% CI(0.62, 2.07), p = 0.692). However, salpingostomy combined with methotrexate showed a lower failure rate compared to salpingostomy alone(OR = 1.130, 95% CI(0.017,2.07), p = 0.692). Network meta-analysis indicated that the salpingostomy combined with methotrexate had the lowest failure rate, while there was no significant difference in failure rates between salpingostomy and methotrexate alone.

*The term used might be a typographical error, but the two terms refer to different entities, which could lead to misinterpretation. in salpingostomy procedures, after the incision is made, the fallopian tube is left open, whereas in salpingotomy, the procedure is followed by suturing of the tube.*

**4. Discussion**

The incidence of tubal pregnancy has been rising globally, accounting for approximately 1–2% of all pregnancies [25]. As a common obstetric emergency, untreated tubal pregnancies may lead to fallopian tube rupture, massive hemorrhage, and life-threatening complications [26]. Current treatment modalities include both conservative and surgical approaches, each associated with varying rates of subsequent intrauterine pregnancy (IUP) and recurrent ectopic pregnancy (REP) depending on the chosen intervention [27].

*The magnitude of incidence has already been mentioned in the introduction, so if it needs to be brought up again, it should be presented briefly.*

Salpingostomy, a fertility-preserving surgical approach, plays a critical role in the management of tubal pregnancies by preserving the affected fallopian tube, thereby maintaining the potential for future natural conception. This approach is particularly vital for patients desiring fertility, especially those with contralateral tubal dysfunction or absence. A prospective study demonstrated significantly higher postoperative spontaneous pregnancy rates in patients undergoing salpingostomy compared to those who underwent salpingectomy [28], underscoring its advantage in preserving reproductive potential. Conversely, other studies reported no significant differences in intrauterine pregnancy (IUP) or reproductive event (REP) rates between salpingostomy and methotrexate (MTX) therapy [29].

Comment :

- *Overgeneralization from a single study* : relying on a single prospective study as the basis for making broad claims about the "advantage in preserving reproductive potential" risks overstatement.
- *Failure to acknowledge potential bias* : A prospective study is not a randomized controlled trial (RCT), so there remains the possibility of confounding factors (e.g., patients with healthier fallopian tubes may have been more likely to undergo salpingostomy). It is not mentioned whether the observed significant difference was statistically adjusted for other influencing variables.
- *The phrase ‘significantly higher’ requires numerical data*
  In academic writing, the term "significantly higher" should ideally be supported by numerical statistics (e.g., p-value, RR/OR, confidence intervals) to allow readers to assess the magnitude and validity of the effect.

Cost-effectiveness analyses indicate that fixed multi-dose MTX protocols are marginally superior to laparoscopic salpingostomy, although this difference is not statistically significant. Multi-dose MTX is considered cost-effective only when serum human chorionic gonadotropin (hCG) concentrations are below 3,000 IU/L, whereas single-dose MTX becomes cost-effective at hCG levels below 1,500 IU/L. Overall, laparoscopic salpingostomy remains the most cost-effective approach [30]. For patients with initial hCG levels between 1,500 and 3,000 IU/L, the costs of salpingostomy and MTX are comparable; however, MTX becomes more expensive when hCG levels exceed 3,000 IU/L [31].

Comment :

*which one is most cost effective (potentially ambiguous)*

- *Cost-effectiveness analyses indicate that fixed multi-dose MTX protocols are marginally superior to laparoscopic salpingostomy*
- *Overall, laparoscopic salpingostomy remains the most cost-effective approach*

Health-related quality of life (HRQoL) studies indicate that systemic methotrexate (MTX) therapy is linked to greater impairments in physical, role, and social functioning, as well as poorer health perception, increased pain, and higher rates of depression compared to surgical management [32].

Comment :

*Does it include an analysis? Does it the conclusion of someone else's research findings? How about your own study?*

Thus, salpingostomy combined with MTX not only reduces failure rates but also minimizes adverse effects on patient well-being.

Comment :

*Logically, multimodal therapy tends to result in more side effects.*

**5. Strengths and Limitations**

"Although the total sample size in this meta-analysis was relatively small due to the limited number of eligible studies available in the databases, this could impact the generalizability of the findings. The inclusion of additional studies with larger sample sizes could help strengthen the conclusions."

**6. Conclusion**

For hemodynamically stable patients with fertility aspirations *(preserving desire),* salpingostomy combined with methotrexate (MTX) represents the optimal therapeutic strategy for tubal pregnancy, offering the lowest failure rate and minimal adverse impact on quality of life compared to MTX monotherapy or salpingostomy alone. These findings underscore the importance of individualized treatment selection based on human chorionic gonadotropin (hCG) levels, cost considerations, and patient-centered outcomes.

*This conclusion is not entirely consistent with the given title, which is* ***Comparison of tubal-preserving surgical management versus methotrexate therapy for tubal pregnancy: a conventional and network meta-analysis.***

*Reasons for the inconsistency:*

*The research title focuses on the comparison between surgical management that preserves the tube (salpingostomy) and methotrexate therapy in the management of tubal pregnancy. Therefore, the core of the study is to assess the effectiveness of treatments based on failure rates and compare these two treatment options.*

*The conclusion, which mentions "individualized treatment selection" and considers hCG levels, costs, and patient-centered outcomes, leans more toward* ***personalized treatment considerations*** *that encompass various factors. This does not fully encapsulate the findings of the study, which primarily focuses on analyzing the failure rates between the two therapy options.*

What should be improved

*To align the conclusion with the main goal of the research, it should emphasize the comparison of failure rates between salpingostomy and methotrexate and the key findings related to their effectiveness, rather than focusing on clinical and practical factors such as costs and hCG levels.*
