# Supplementary material for: Comparison of laparoscopic tubal-preserving surgical management versus methotrexate therapy for tubal pregnancy: A conventional and network meta-analysis
Source: PLoS One. 2025 Sep 12;20(9):e0332169. doi: 10.1371/journal.pone.0332169 (PMC12431245; doi:10.1371/journal.pone.0332169)
Supplement: Attachment — Submitted filename: Response to Reviewers.docx [file pone.0332169.s003.docx]

Response to Reviewers

Journal Requirements:

When submitting your revision, we need you to address these additional requirements.

1. Please ensure that your manuscript meets PLOS ONE's style requirements, including those for file naming. The PLOS ONE style templates can be found at

[https://journals.plos.org/plosone/s/file?id=wjVg/PLOSOne_formatting_sample_main_body.pdf](https://journals.plos.org/plosone/s/file?id=wjVg/PLOSOne_formatting_sample_main_body.pdf) and

[https://journals.plos.org/plosone/s/file?id=ba62/PLOSOne_formatting_sample_title_authors_affiliations.pdf](https://track.editorialmanager.com/CL0/https://journals.plos.org/plosone/s/file?id=ba62/PLOSOne_formatting_sample_title_authors_affiliations.pdf/1/010f01981373ad7a-1fc0943e-0e9a-4a69-8e8d-70f4831545a2-000000/MZ9kj9-kIkpJkAbYPp8NKcp4SM0G_GqipGwnOwoVh38=219)

2. Thank you for stating the following in the Competing Interests section:
   The authors claim there is no conflict of interest

   Please confirm that this does not alter your adherence to all PLOS ONE policies on sharing data and materials, by including the following statement: "This does not alter our adherence to PLOS ONE policies on sharing data and materials.” (as detailed online in our guide for authors [http://journals.plos.org/plosone/s/competing-interests](http://journals.plos.org/plosone/s/competing-interests)). If there are restrictions on sharing of data and/or materials, please state these. Please note that we cannot proceed with consideration of your article until this information has been declared.

   Please include your updated Competing Interests statement in your cover letter; we will change the online submission form on your behalf.

**Answer:Modified.**

7.2 Competing Interests

The authors claim there is no conflict of interest. The authors have no relevant financial or non-financial interests to disclose. This does not alter our adherence to PLOS ONE policies on sharing data and materials.

3. Please ensure that you refer to Figures 5 and 6 in your text as, if accepted, production will need this reference to link the reader to the figures.

**Answer:Modified.**

3.6 Publication Bias

The funnel plots of the included studies appeared to be roughly symmetrical. Additionally, we conducted Begg's test and Egger's test to assess the presence of publication bias in this study, and no significant publication bias was detected. This result is shown in Figure 5 and 6.

4. Please remove all personal information, ensure that the data shared are in accordance with participant consent, and re-upload a fully anonymized data set.

Note: spreadsheet columns with personal information must be removed and not hidden as all hidden columns will appear in the published file.

Additional guidance on preparing raw data for publication can be found in our Data Policy ([https://journals.plos.org/plosone/s/data-availability#loc-human-research-participant-data-and-other-sensitive-data](https://journals.plos.org/plosone/s/data-availability#loc-human-research-participant-data-and-other-sensitive-data)) and in the following article: [http://www.bmj.com/content/340/bmj.c181.long](http://track.editorialmanager.com/CL0/http://www.bmj.com/content/340/bmj.c181.long/1/010f01981373ad7a-1fc0943e-0e9a-4a69-8e8d-70f4831545a2-000000/G3VkuudFD_lpofiEMfyo85I88mID8EjSiAzSo6O1OS4=219).

5. Please include captions for your Supporting Information files at the end of your manuscript, and update any in-text citations to match accordingly. Please see our Supporting Information guidelines for more information: [http://journals.plos.org/plosone/s/supporting-information.](http://journals.plos.org/plosone/s/supporting-information.)

**Answer:Modified.**

S1 Table Results of the evidence quality assessment of the studies

6. If the reviewer comments include a recommendation to cite specific previously published works, please review and evaluate these publications to determine whether they are relevant and should be cited. There is no requirement to cite these works unless the editor has indicated otherwise.

Additional Editor Comments:

This munuscript is quite interesting, but there are some issues that must be addressed.

First, regarding study inclusion, in addition to the factors already listed by the authors, geographic region and ethnicity are also critically important, as they directly impact treatment effectiveness.

**Answer:Modified.**

| Study | geographic region | ethnicity | Study type | age | | Sample | | Gestational sac diameter | | average HCG | |
| --- | --- | --- | --- | --- | --- | --- | --- | --- | --- | --- | --- |
| Salpingostomy combined with MTX/MTX | | | | | | | | | | | |
| J W Graczykowski 1997 | Los Angeles, USA | - | RCT | 27.9±5.3 | 26.2±4.4 | 54 | 62 | - | - | - | - |
| Shigeo Akira 2008 | Tokyo | - | controlled trial | 29.2±5.3 | 29.8 ±4.0 | 41 | 40 | - | - | 3287 ± 3230 (250-13012) | 3165±2758 (198-12756) |
| Hakan Kaya, M 2002 | Isparta ,Turkey | - | RCT | 28.8± 5.2 | 30.1±5.8 | 22 | 43 | 2.4±0.9 | 2.3±1.0 | 245-7800 | 450-7200 |
| Methotrexate/salpingostomy | | | | | | | | | | | |
| P J Hajenius 1997 | Amsterdam, Netherlands | - | RCT | 31.3±5.9 | 31.8±4.4 | 51 | 49 | - | - | 1950(110-  19500) | 2100(228-  18400) |
| F.Lecuru 1998 | Paris, France | - | controlled trial | 31 (17-45) | 29 (19-38) | 37 | 38 | - | - | 585(50-5525) | 998(98-  6000) |
| ASMA J. SARAJ 1998 | Los Angeles, USA | - | RCT | 28.1± 1.2 | 27.4 ± 1.0 | 38 | 36 | 2.7±0.1 | 2.7± 0.3 | 3162±772 | 3357±766 |
| Martin C. Sowter 2001 | Auckland, New Zealand |  | RCT | 29.7± 5.4 | 30.4 ±4.6 | 34 | 26 | - | - | 927  (137 - 4866) | 775 (89 - 4800) |
| Lars Bo Krag Moeller 2009 | Hvidovre, Denmark | - | RCT | 30.8 (21.1–40.9) | 32.7 (24.3–41.2) | 53 | 53 | 1.2 (0.5-2.5) | 1.4 (0.7-2.9) | 2259 (176-4100) | 3200 (72-42859) |

Second, the figures and tables in the article suffer from inconsistent font styles and cluttered formatting, which need to be improved for better readability and presentation.
 **Answer:Modified.**

Third, in the Discussion section, much of the current content would be more appropriately placed in the Introduction. The discussion should instead focus on your findings and their interpretation.
 **Answer:Modified.**

4. Discussion

Salpingostomy, a fertility-preserving surgical approach, plays a critical role in the management of tubal pregnancies by preserving the affected fallopian tube, thereby maintaining the potential for future natural conception. This approach is particularly vital for patients desiring fertility, especially those with contralateral tubal dysfunction or absence. A prospective study demonstrated higher(OR = 1.61, 95% CI: 1.29-2.01) postoperative spontaneous pregnancy rates in patients undergoing salpingostomy compared to those who underwent salpingectomy [28], underscoring its advantage in preserving reproductive potential. Conversely, other studies reported no significant differences in intrauterine pregnancy (IUP) or repeat ectopic pregnancy (REP) rates between salpingostomy and methotrexate (MTX) therapy [29].

MTX, a folate antagonist, terminates pregnancy by inhibiting cellular proliferation and disrupting trophoblastic cells. Single-dose MTX regimens are widely recognized as safe and effective for hemodynamically stable patients with early-diagnosed ectopic pregnancies [28].

However, our analysis shows that in general, the failure rate of methotrexate (MTX) is higher than that of salpingostomy ; while the subgroup analysis indicates that the failure rate of single-dose MTX treatment is higher than that of salpingostomy, and the failure rate of the double-dose MTX regimen does not show a significant difference compared to salpingostomy. These suggest that a single-dose intervention with methotrexate may be associated with a higher risk of treatment failure, while two doses administrations do not show a significant difference, indicating that in clinical practice, it may be necessary to optimize the methotrexate administration regimen (such as increasing the dose) to reduce the failure rate, but this needs further verification.

Our meta-analysis shows also that the failure rate of salpingostomy combined with methotrexate treatment for tubal pregnancy is significantly lower than that of simple salpingostomy. Through network meta-analysis, it is further confirmed that salpingostomy combined with MTX treatment is the most effective, and compared with methotrexate and simple salpingostomy, salpingostomy combined with MTX has a lower failure rate while preserving the function of the fallopian tube.

According to our meta-analysis results, when hemodynamics were stable, we observed that the levels of blood HCG on days 4 and 7 following the first treatment with methotrexate were unsatisfactory, that is, the decrease in blood HCG levels is less than 15%. If hemodynamics were still stable, we could inject methotrexate again to treat the tubal pregnancy. If the patient wishes to leave the hospital as soon as possible, a salpingostomycombined with methotrexate treatment for the tubal pregnancy is undoubtedly a good choice.

Cost-effectiveness analyses indicate that laparoscopic salpingostomy remains the most cost-effective approach. Multi-dose MTX is considered cost-effective only when serum human chorionic gonadotropin (hCG) concentrations are below 3,000 IU/L, whereas single-dose MTX becomes cost-effective at hCG levels below 1,500 IU/L[30]. For patients with initial hCG levels between 1,500 and 3,000 IU/L, the costs of salpingostomy and MTX are comparable; however, MTX becomes more expensive when hCG levels exceed 3,000 IU/L [31].

Health-related quality of life (HRQoL) studies indicate that systemic methotrexate (MTX) therapy is linked to greater impairments in physical, role, and social functioning, as well as poorer health perception, increased pain, and higher rates of depression compared to surgical management [32]. Though salpingostomy combined with MTX reduces failure rates, multimodal therapy may be result in more side effects.

Although the two reviewers did not raise many concerns, I still strongly recommend that this manuscript undergo thorough revision.


[Note: HTML markup is below. Please do not edit.]

Reviewers' comments:

Reviewer's Responses to Questions

**Comments to the Author**

1. Is the manuscript technically sound, and do the data support the conclusions?

The manuscript must describe a technically sound piece of scientific research with data that supports the conclusions. Experiments must have been conducted rigorously, with appropriate controls, replication, and sample sizes. The conclusions must be drawn appropriately based on the data presented.

Reviewer #1: Yes

Reviewer #2: Yes

2. Has the statistical analysis been performed appropriately and rigorously?

Reviewer #1: Yes

Reviewer #2: Yes

3. Have the authors made all data underlying the findings in their manuscript fully available?

The [PLOS Data policy](http://track.editorialmanager.com/CL0/http://www.plosone.org/static/policies.action%23sharing/1/010f01981373ad7a-1fc0943e-0e9a-4a69-8e8d-70f4831545a2-000000/2NF16117aLWG4-UKuXArEKI44-qYTxuYF8F3CEI8NKs=219) requires authors to make all data underlying the findings described in their manuscript fully available without restriction, with rare exception (please refer to the Data Availability Statement in the manuscript PDF file). The data should be provided as part of the manuscript or its supporting information, or deposited to a public repository. For example, in addition to summary statistics, the data points behind means, medians and variance measures should be available. If there are restrictions on publicly sharing data—e.g. participant privacy or use of data from a third party—those must be specified.

Reviewer #1: Yes

Reviewer #2: Yes

4. Is the manuscript presented in an intelligible fashion and written in standard English?

PLOS ONE does not copyedit accepted manuscripts, so the language in submitted articles must be clear, correct, and unambiguous. Any typographical or grammatical errors should be corrected at revision, so please note any specific errors here.

Reviewer #1: Yes

Reviewer #2: No

5. Review Comments to the Author

Please use the space provided to explain your answers to the questions above. You may also include additional comments for the author, including concerns about dual publication, research ethics, or publication ethics. (Please upload your review as an attachment if it exceeds 20,000 characters)

Reviewer #1: I thank the author for taking out time to carryout this very important piece of work.

There are few queries I would want the authors to adress
1. There are continuing concerns with recurrent ectopic pregnancy following salpingotomy/salpingostomy due to scarring. Kindly address this.

**Answer:Modified.**

The incidence of tubal pregnancy has been rising globally, accounting for approximately 1–2% of all pregnancies [3]. As a common obstetric emergency, untreated tubal pregnancies may lead to fallopian tube rupture, massive hemorrhage, and life-threatening complications [4]. Current treatment modalities include both conservative and surgical approaches, each associated with varying rates of subsequent intrauterine pregnancy (IUP) and recurrent ectopic pregnancy (REP) depending on the chosen intervention [5].

2. In selecting the works on medical management of tubal pregnancy, did you consider the size of the ectopic mass? If yes, what was the eligible maximum diameter for medical management. This is very important as this, in addition to the level of hCG, is a determinant of success rate.
 **Answer:Modified.**

| Study | geographic region | ethnicity | Study type | age | | Sample | | Gestational sac diameter(cm) | | average HCG | |
| --- | --- | --- | --- | --- | --- | --- | --- | --- | --- | --- | --- |
| Salpingostomy combined with MTX/MTX | | | | | | | | | | | |
| J W Graczykowski 1997 | Los Angeles, USA | - | RCT | 27.9±5.3 | 26.2±4.4 | 54 | 62 | - | - | - | - |
| Shigeo Akira 2008 | Tokyo | - | controlled trial | 29.2±5.3 | 29.8 ±4.0 | 41 | 40 | - | - | 3287 ± 3230 (250-13012) | 3165±2758 (198-12756) |
| Hakan Kaya, M 2002 | Isparta ,Turkey | - | RCT | 28.8± 5.2 | 30.1±5.8 | 22 | 43 | 2.4±0.9 | 2.3±1.0 | 245-7800 | 450-7200 |
| Methotrexate/salpingostomy | | | | | | | | | | | |
| P J Hajenius 1997 | Amsterdam, Netherlands | - | RCT | 31.3±5.9 | 31.8±4.4 | 51 | 49 | - | - | 1950(110-  19500) | 2100(228-  18400) |
| F.Lecuru 1998 | Paris, France | - | controlled trial | 31 (17-45) | 29 (19-38) | 37 | 38 | - | - | 585(50-5525) | 998(98-  6000) |
| ASMA J. SARAJ 1998 | Los Angeles, USA | - | RCT | 28.1± 1.2 | 27.4 ± 1.0 | 38 | 36 | 2.7±0.1 | 2.7± 0.3 | 3162±772 | 3357±766 |
| Martin C. Sowter 2001 | Auckland, New Zealand |  | RCT | 29.7± 5.4 | 30.4 ±4.6 | 34 | 26 | - | - | 927  (137 - 4866) | 775 (89 - 4800) |
| Lars Bo Krag Moeller 2009 | Hvidovre, Denmark | - | RCT | 30.8 (21.1–40.9) | 32.7 (24.3–41.2) | 53 | 53 | 1.2 (0.5-2.5) | 1.4 (0.7-2.9) | 2259 (176-4100) | 3200 (72-42859) |

3. Justify your use of decrease of less than 20% on days 4 & 7 as your definition of failure of medical management. A change of <15% between the day 4 and 7 values is widely used and we know that it is usual not to have a significant decrease or even an increase level on day 4 level following medical management.

**Answer:Modified.**

Methotrexate treatment failure is defined as a reduction of less than 15% in the serum human chorionic gonadotropin (HCG) levels on days 4 and 7 following methotrexate administration in cases of tubal pregnancy.

4. You appear to interchange the use salpingotomy and salpingostomy eventhough they are different procedures. Can you rectify this please.

**Answer:The entire text has been revised.**

Reviewer #2: While the manuscript generally reflects scientific research that supports its conclusions, some parts of the discussion—particularly those addressing quality of life and cost—lack accompanying data analysis. The conclusions in these sections seem to be derived from studies conducted by other researchers.

**Answer:Modified.**

Cost-effectiveness analyses indicate that laparoscopic salpingostomy remains the most cost-effective approach. Multi-dose MTX is considered cost-effective only when serum human chorionic gonadotropin (hCG) concentrations are below 3,000 IU/L, whereas single-dose MTX becomes cost-effective at hCG levels below 1,500 IU/L[30]. For patients with initial hCG levels between 1,500 and 3,000 IU/L, the costs of salpingostomy and MTX are comparable; however, MTX becomes more expensive when hCG levels exceed 3,000 IU/L [31].

Health-related quality of life (HRQoL) studies indicate that systemic methotrexate (MTX) therapy is linked to greater impairments in physical, role, and social functioning, as well as poorer health perception, increased pain, and higher rates of depression compared to surgical management [32]. Though salpingostomy combined with MTX reduces failure rates, multimodal therapy may be result in more side effects.

6. PLOS authors have the option to publish the peer review history of their article ([what does this mean?](https://track.editorialmanager.com/CL0/https://journals.plos.org/plosone/s/editorial-and-peer-review-process%23loc-peer-review-history/1/010f01981373ad7a-1fc0943e-0e9a-4a69-8e8d-70f4831545a2-000000/eLUVSB_KazVxJpdAoMV-p5Bv056uAjF5lQPhoSFTJGE=219)). If published, this will include your full peer review and any attached files.

If you choose “no”, your identity will remain anonymous but your review may still be made public.

**Do you want your identity to be public for this peer review?** For information about this choice, including consent withdrawal, please see our [Privacy Policy](https://track.editorialmanager.com/CL0/https://www.plos.org/privacy-policy/1/010f01981373ad7a-1fc0943e-0e9a-4a69-8e8d-70f4831545a2-000000/8bojxdt6TCLQhPoUiEH5cNpJP22lugeiF9eUdYBZGwg=219).

Reviewer #1: No

Reviewer #2: No

[NOTE: If reviewer comments were submitted as an attachment file, they will be attached to this email and accessible via the submission site. Please log into your account, locate the manuscript record, and check for the action link "View Attachments". If this link does not appear, there are no attachment files.]
