# Supplementary material for: Comparison of laparoscopic tubal-preserving surgical management versus methotrexate therapy for tubal pregnancy: A conventional and network meta-analysis
Source: PLoS One. 2025 Sep 12;20(9):e0332169. doi: 10.1371/journal.pone.0332169 (PMC12431245; doi:10.1371/journal.pone.0332169)
Supplement: Attachment — Submitted filename: fix Re-review PLOS.docx [file pone.0332169.s004.docx]

Abstract

Background

Tubal pregnancy is one of the common emergencies in obstetrics and gynecology. There are various treatment options for tubal pregnancy. However, there is no consensus on how patients should choose among the treatment options that preserve the fallopian tube. This study aims to investigate the prognostic outcomes of different treatment modalities for tubal pregnancy while preserving the fallopian tube.

*Comment : Slight redundancy in "treatment modalities for tubal pregnancy while preserving the fallopian tube" — all treatment options discussed already aim to preserve the tube.*

*Suggestion : consider rephrasing to avoid repetition*

Methods
Databases, including the Cochrane Library, EMBASE, and PubMed, were searched up to February 2025. Statistical analysis was performed using Stata 17.0 software.

*Comment : Not sufficiently informative, even for the methods section of the abstract.*

Result

A total of 8 studies involving 677 patients were included. Meta-analysis revealed a significant overall difference in the failure rate between methotrexate treatment
and salpingostomy for tubal pregnancy(OR=1.582, 95%CI(1.062, 2.358), p = 0.024). Subgroup analysis demonstrated that a single dose of methotrexate was associated with a higher failure rate compared to salpingostomy(OR = 2.044,95%CI(1.20, 3.47), p = 0.008). In contrast, two or more doses of methotrexate did not show a significant difference in failure rate compared to salpingostomy(OR = 1.130, 95% CI(0.62, 2.07), p = 0.692). **However, salpingostomy combined with methotrexate showed a lower failure rate compared to salpingostomy alone(OR = 0.11, 95% CI (0.03-0.48), p = 0.692).** Network meta-analysis indicated that the salpingostomy combined with methotrexate had the lowest failure rate, while there was no significant difference in failure rates between salpingostomy and methotrexate alone.

*Comment :*

*Contradictory p-value in Combined Treatment Result*

*The OR and 95% CI suggest a statistically significant difference, but the p-value of 0.692 indicates no significance, could clarify whether methotrexate is used pre-, intra-, or post-operatively, as that affects interpretation. it would be helpful to define what constitutes "failure" in this context.*

**Fulltext**

Background

Ectopic pregnancy (EP) is defined as a pregnancy occurring outside the uterine cavity, most commonly in the fallopian tubes (96%) [1]. Tubal pregnancy, a subtype of ectopic pregnancy, refers to the implantation and development of a fertilized ovum within the fallopian tube. Under normal physiological conditions, the fertilized ovum traverses the fallopian tube and implants in the endometrial lining. However, in tubal pregnancies, impaired tubal function—caused by factors such as inflammation, adhesions, surgical history, or congenital anomalies—prevents the embryo from migrating to the uterus, resulting in ectopic implantation. Tubal pregnancies account for over 95% of all ectopic pregnancies, making them the most prevalent form [2].

*Comment:*

*Redundancy : streamline these into one clear definition and prevalence of tubal pregnancy, for example tubal pregnancy, accounting for over 95% of ectopic pregnancies, refers to the implantation of a fertilized ovum within the fallopian tube."*

The incidence of tubal pregnancy has been rising globally, accounting for approximately 1–2% of all pregnancies [3]. As a common obstetric emergency, untreated tubal pregnancies may lead to fallopian tube rupture, massive hemorrhage, and life-threatening complications [4]. Current treatment modalities include both conservative and surgical approaches, each associated with varying rates of subsequent intrauterine pregnancy (IUP) and recurrent ectopic pregnancy (REP) depending on the chosen intervention [5].

Treatment options for tubal pregnancy vary and include expectant management, methotrexate (MTX) therapy, salpingectomy, and salpingostomy. Studies indicate that MTX treatment does not achieve a 100% success rate in hemodynamically stable EP patients [6-8]. Similarly, salpingostomy carries a risk of retained trophoblastic tissue, which may necessitate adjuvant medical therapy or repeat surgical intervention [9,10]. While growing attention has been directed toward reproductive outcomes in EP patients, particularly those desiring future fertility, treatment failure rates remain a critical concern due to their direct impact on patient recovery time and healthcare costs [11-13].

*Comments: Lack of clear definition of “failure rate”*

**Notably, salpingostomy has been reported to be a cost-effective approach compared to medical therapy [14].**

*Comments:*  ***Bold : this could be bias the reader before the data is presented. This could be a premature conclusion between mtx and salpingectomy***

**Therefore, clarifying the failure rates associated with different treatment modalities for tubal pregnancy to guide optimal therapeutic decision-making**. “

*Comment : this could be fragment sentence without main verb, ‘it is important to clarify..’*

This meta-analysis reviews the existing literature comparing failure rates of methotrexate (MTX), salpingostomy, and salpingostomy combined with MTX in the management of tubal pregnancy, aiming to evaluate their prognostic outcomes and inform clinical practice.

2.1 Inclusion and Exclusion Criteria

Inclusion criteria: 1. Study designs: Randomized controlled trials (RCTs), observational studies, comparative studies, clinical trials, multicenter studies, or controlled studies. 2. Study population: Hemodynamically stable women diagnosed with tubal pregnancy. 3. Interventions: Treatment with methotrexate (MTX) or salpingostomy. 4. Outcome measures: Inclusion of treatment failure rate as a primary outcome.

*Comments:*

- *Study Population : reproductive age? Why need to preserve tubal if they don’t*
- *Treatment : Combination included?*

Exclusion criteria: 1. Non-tubal ectopic pregnancies (e.g., ovarian or cervical pregnancies). 2. Ultrasonographic evidence of tubal rupture or hemoperitoneum. 3. Animal studies, cell studies, reviews, meta-analyses, case reports, letters, or duplicate publications. 4. Studies with incomplete data, methodological errors, or outcomes inconsistent with inclusion criteria.

*Comment : need to elaborate term, “ incomplete data”*

2.2 Search Strategy

A comprehensive search was conducted in PubMed, EMbase, Web of Science, and the Cochrane Library from the inception of these databases to February 2025. Free search terms included combinations of "tubal pregnancy," "ectopic pregnancy," and "salpingostomy". Two researchers independently performed the literature search and manual screening. Any discrepancies were resolved through consultation with a third researcher.

*Comment :* ***Did not include a complete search strategy or Boolean keywords.*** *Is there language restriction such as non english publication or ongoing trial?*

2.3 Data Extraction

Two investigators (Yong Lin and Junwei Liao) independently screened and extracted data from eligible studies. Any disagreements were adjudicated by a third author (Yan Long). Extracted data included: 1. Study characteristics: First author, publication year, study design, sample size, and participant age. 2. Interventions: Methotrexate (MTX), salpingostomy, or combined therapy. 3. Clinical parameters: Baseline serum hCG levels, gestational sac size, and duration of follow-up. 4. Outcomes: Treatment failure rate.

Methotrexate treatment failure is defined as a reduction of less than 15% in the serum human chorionic gonadotropin (HCG) levels on days 4 and 7 following methotrexate administration in cases of tubal pregnancy.

.5 Quality Assessment

The quality was evaluated using the Newcastle-Ottawa Scale (NOS) for controlled studies and the modified Jadad scale for RCTs. NOS assesses three domains: selection (0–4 points), comparability (0–2 points), and outcome/exposure (0–3 points). Total scores categorize studies as low (0–3), moderate (4–6), or high quality (7–9) [15]. Modified Jadad scale evaluates randomized controlled trials (RCTs) on a 7-point scale (low quality: 1–3; high quality: 4–7) [16]. Missing data were requested from the corresponding authors. Any discrepancies were resolved via consensus or third researcher.

***Comment :*** *"Did not mention whether other risk of bias assessments were conducted."*

**4. discussion**

Salpingostomy, a fertility-preserving surgical approach, plays a critical role in the management of tubal pregnancies by preserving the affected fallopian tube, thereby maintaining the potential for future natural conception. This approach is particularly vital for patients desiring fertility, especially those with contralateral tubal dysfunction or absence. A prospective study demonstrated higher(OR = 1.61, 95% CI: 1.29-2.01) postoperative spontaneous pregnancy rates in patients undergoing salpingostomy compared to those who underwent salpingectomy [28], underscoring its advantage in preserving reproductive potential. Conversely, other studies reported no significant differences in intrauterine pregnancy (IUP) or repeat ectopic pregnancy (REP) rates between salpingostomy and methotrexate (MTX) therapy [29].

MTX, a folate antagonist, terminates pregnancy by inhibiting cellular proliferation and disrupting trophoblastic cells. Single-dose MTX regimens are widely recognized as safe and effective for hemodynamically stable patients with early-diagnosed ectopic pregnancies [28].

**Comment:**

- *The description of the function of salpingostomy and the observational study comparing salpingostomy with salpingectomy are more appropriately placed in the Introduction section, as they are not findings from your own study and are not directly compared in your network meta-analysis.*

However, our analysis shows that in general, the failure rate of methotrexate (MTX) is higher than that of salpingostomy ; while the subgroup analysis indicates that the failure rate of single-dose MTX treatment is higher than that of salpingostomy, and the failure rate of the double-dose MTX regimen does not show a significant difference compared to salpingostomy. These suggest that a single-dose intervention with methotrexate may be associated with a higher risk of treatment failure, while two doses administrations do not show a significant difference, indicating that in clinical practice, it may be necessary to optimize the methotrexate administration regimen (such as increasing the dose) to reduce the failure rate, but this needs further verification.

Our meta-analysis shows also that the failure rate of salpingostomy combined with methotrexate treatment for tubal pregnancy is significantly lower than that of simple salpingostomy. Through network meta-analysis, it is further confirmed that salpingostomy combined with MTX treatment is the most effective, and compared with methotrexate and simple salpingostomy, salpingostomy combined with MTX has a lower failure rate while preserving the function of the fallopian tube.

According to our meta-analysis results, when hemodynamics were stable, we observed that the levels of blood HCG on days 4 and 7 following the first treatment with methotrexate were unsatisfactory, that is, the decrease in blood HCG levels is less than 15%. If hemodynamics were still stable, we could inject methotrexate again to treat the tubal pregnancy. If the patient wishes to leave the hospital as soon as possible, a salpingostomycombined with methotrexate treatment for the tubal pregnancy is undoubtedly a good choice.

*Comment : the statement “salpingostomy combined with methotrexate treatment for the tubal pregnancy is undoubtedly a good choice” uses somewhat exaggerated or overly emphatic language*

Cost-effectiveness analyses indicate that laparoscopic salpingostomy remains the most cost-effective approach. Multi-dose MTX is considered cost-effective only when serum human chorionic gonadotropin (hCG) concentrations are below 3,000 IU/L, whereas single-dose MTX becomes cost-effective at hCG levels below 1,500 IU/L[30]. For patients with initial hCG levels between 1,500 and 3,000 IU/L, the costs of salpingostomy and MTX are comparable; however, MTX becomes more expensive when hCG levels exceed 3,000 IU/L [31].

Health-related quality of life (HRQoL) studies indicate that systemic methotrexate (MTX) therapy is linked to greater impairments in physical, role, and social functioning, as well as poorer health perception, increased pain, and higher rates of depression compared to surgical management [32]. Though salpingostomy combined with MTX reduces failure rates, multimodal therapy may be result in more side effects.

*Comment : Although the lack of information regarding the route of salpingostomy—whether performed via laparoscopy or laparotomy—does not undermine your study as a whole, considering that you mention quality of life and cost, wouldn’t it be necessary to provide some explanation regarding this aspect?*
