# Supplementary material for: Comparison of laparoscopic tubal-preserving surgical management versus methotrexate therapy for tubal pregnancy: A conventional and network meta-analysis
Source: PLoS One. 2025 Sep 12;20(9):e0332169. doi: 10.1371/journal.pone.0332169 (PMC12431245; doi:10.1371/journal.pone.0332169)
Supplement: Attachment — Submitted filename: Response to Reviewers(1).docx [file pone.0332169.s005.docx]

Response to Reviewers

6. Review Comments to the Author

Please use the space provided to explain your answers to the questions above. You may also include additional comments for the author, including concerns about dual publication, research ethics, or publication ethics. (Please upload your review as an attachment if it exceeds 20,000 characters)

Reviewer #1: I wish to thank the authors for taking out the time to address the queries. I have no further concerns.

Reviewer #2: Considering that not all hospitals have adequate resources and facilities, particularly for laparoscopic procedures, it would be advisable to address this aspect in the manuscript. While this may not substantially alter or diminish the overall value of your publication, it is nonetheless a point worth noting.

**Answer:There are few literature on non-laparoscopic surgical treatment methods such as open surgery. In the future, we will systematically evaluate whether there is a difference in the efficacy of different treatment plans when more data are collected**
